# Adjudication of the Alleged Role of Vitamin D in the Antimicrobial Pathway

**DOI:** 10.6064/2012/129516

**Published:** 2012-06-25

**Authors:** Gerald M. Higa, Jason Hicks, Christopher Isabella

**Affiliations:** ^1^Schools of Pharmacy and Medicine and the Mary Babb Randolph Cancer Center, West Virginia University, Morgantown, WV 26506, USA; ^2^School of Pharmacy, West Virginia University, Morgantown, WV 26506, USA

## Abstract

Dynamic interactions between microorganism and host have evolved in such a way that while microbial pathogens are the cause of many human infections, a symbiotic relationship is also known to exist. Another important anomaly is that exposure to pathogenic organisms does not necessarily result in development of clinical disease. The latter conclusion infers that susceptibility to infectious disease can be modified by host-related factors. Arguably the two most prominent factors are genetic variability and immunologic status of the exposed individual. Because of the Human Genome and the HapMap projects, developments in genotyping technology have brought the possibility of identifying associations between specific genetic alterations and common diseases closer to reality. In addition, a growing body of evidence suggests vitamin D has an important contributory role in the antimicrobial pathway.

## 1. Introduction

Epidemiologic, biologic, and even genotypic data convincingly demonstrate the essential role of microorganisms in the genesis of many human infections; even so, exposure alone does not necessarily result in development of clinical disease. Inherent in this technically accurate paradox is that while the former supports the well-established link between microbial pathogens and infectious diseases, the latter infers that disease susceptibility can be modified by extramicrobial factors [1–3]. Arguably, the most important “other” factor is adaptive and innate immunity of the exposed individual. Furthermore, accumulating data indicate that alterations of immune responsiveness are partly dependent on genetic factors [4]. For example, in addition to the one gene-one infection paradigm such as the Duffy-null genotype, Fy^a-b-^ (the most common genotype among those of black African heritage), and resistance to malaria [5], there is also evidence which supports the association between one gene with multiple types of infections or multiple genes with a single type of infection [6]. Hence, susceptibility to infection can be characterized by a construct that involves not only gene expression patterns of the host but also that of the microorganism, both of which continue to adapt in response to external stimuli. A plausible extension of this concept relates to the involvement of vitamin D in immunomodulation and the role the vitamin appears to have in a number of infectious diseases.

**Figure 1:**
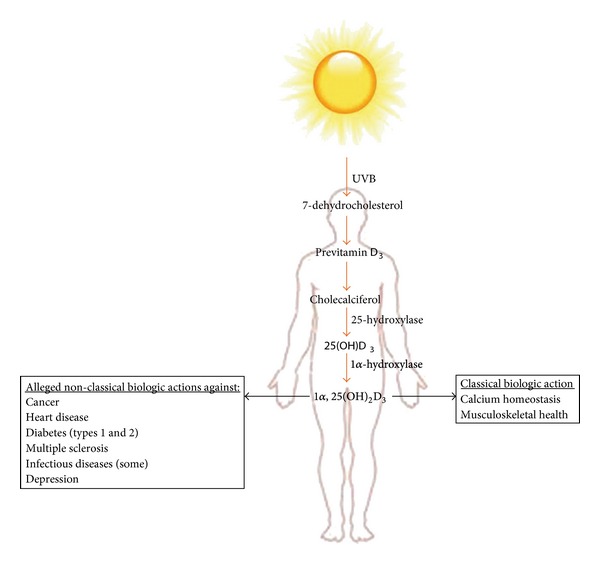
Synthesis and biologic actions of vitamin D_3_. A number of nontraditional roles are linked to adverse consequences of vitamin D deficiency.

These preparatory comments notwithstanding, it seems counter-intuitive to suggest that results of laboratory and clinical research can actually increase the uncertainty of what is already a controversy in medicine. Yet, this conclusion is apparently true for vitamin D. Now, perhaps even more than nearly a century ago when the first investigational studies were conducted to elucidate the etiology of rickets [7], research has revealed that the beneficial effects of the vitamin D endocrine system may extend beyond the musculoskeletal system. Indeed, a profound holistic effect on well-being has been linked to a number of nonclassic actions of the vitamin (Figure 1) [8, 9]. This paper focuses on our increased, though by no means complete, understanding of the vitamin-D-dependent antimicrobial pathway and attempts to adjudicate published evidence regarding the vitamin's alleged role in altering host susceptibility to infections.

## 2. Vitamin D Status and Health

Although the Endocrine Society strongly recommends using serum 25(OH)D levels to classify vitamin D status in humans [10] there is still some inconsistency regarding the cut-off point that distinguishes sufficient from insufficient levels of the vitamin. Even the recommended sufficient range of 25–80 ng/mL, which was extracted from previous data that did not account for sun exposure, race, age, and geographic latitude, may not necessarily apply to the noncalcemic effects for which the vitamin purportedly has a biological role. Nonetheless, there is evidence, when collectively analyzed, which suggests an association between vitamin D and risk of dying from any cause. One such meta-analysis involved over 57,000 subjects, the majority of who were postmenopausal women, examined the potential benefits of vitamin D supplementation [11]. Among those in the vitamin-supplemented intervention groups, serum 25(OH)D levels were up to five-fold higher compared to the control group. Over an adjusted mean followup of nearly six years 4,777 deaths occurred. Despite daily doses of supplemental vitamin D, which ranged between 300 international units (IUs) and 2,000 (IU), the intervention groups had a 7% reduction in relative risk of dying from any cause compared to the control groups. Although the association between low vitamin D levels and mortality was not statistically significant, the relatively small risk reduction may nevertheless be clinically relevant as deaths due to cardiovascular- and diabetes-related complications (diseases that may be impacted by low vitamin D) claim the lives of approximately 435,000 American women annually [12].

Because serum levels appear to be correlated with physiological and pathological musculoskeletal outcomes [13], it is tempting to speculate that the vitamin's effect in the antimicrobial pathway would also depend merely on maintaining sufficient levels of 25(OH)D. While this belief may have some validity, it also grossly oversimplifies an immensely intricate pharmacological process. And in order to enhance reader appreciation of this complex phenomenon, an overview of the basic biology and molecular pharmacology of vitamin D is presented.

## 3. Basic Biology of Vitamin D

Plant-derived ergosterol and dermal-derived 7-dehydrocholesterol are the precursors of ergocalciferol (D_2_) and cholecalciferol (D_3_), respectively, the two major forms of vitamin D. However, most of the body's vitamin requirement is acquired through ultraviolet B sunlight-cleavage of 7-dehydrocholesterol resulting in D_3_, the predominant, though biologically inert, isoform of the vitamin. Conversion to the metabolically active hormone, 1*α*,25(OH)_2_D_3_, requires two hydroxylation steps, first in the liver and then the kidney. Although some uncertainty still exists regarding the specific enzymes involved in the bioactivation process, cytochrome (CY) P2R1 has been shown to possess very potent hepatic 25-hydroxylase activity [14] while CYP27B1 appears to be the primary renal 1*α*-hydroxylase [15, 16].

The site-specific hydroxylation reactions differ in one major way. While hepatic production of 25(OH)D is partially, and inversely, influenced by serum vitamin D levels, renal activation to the bioactive hormone is a stringently regulated process. This disparity may also explain a pharmacologic inconsistency related to the metabolite that mediates vitamin D toxicity. Instead of the bioactive hormone, it has been reported that the 25-hydroxy metabolite appears to mediate the toxic manifestations [17]. This finding is somewhat surprising because 25(OH)D exhibits two features that would suggest, otherwise, (1) a very high affinity for vitamin-D-binding protein (VDBP) and (2) a low affinity for the vitamin D receptor (VDR). Using CYP27B1^−/−^ (knockout) mice, which do not have the capacity to produce the biologically active hormone, Deluca and colleagues observed similar degrees of vitamin D intoxication in both null and wild-type mice given large doses of D_3_. Severe toxic hypercalcemia occurred with serum levels of 25(OH)D ranging between 800 to 1000 ng/mL. Even more interesting was the finding that while 25(OH)D is biologically inactive at normal serum concentrations, gene assays revealed that, at levels of 400 ng/mL, the metabolite became transcriptionally active. This finding is consistent with other *in vitro* data showing that at high concentrations, the bioactivity of 25(OH)D_3_ mimics 1*α*,25(OH)_2_D_3_ [18]. 

## 4. Molecular Pharmacology of Vitamin D

Conversion of D_3_ to 1*α*,25(OH)_2_D_3_ is a relatively simple process compared to translation of the hormone's effects which involves a number of intricate steps and additional factors. Since a high percentage of circulating steroid hormones are bound to soluble proteins, it is well appreciated that extracellular factors can influence the vitamin's pharmacokinetic and pharmacodynamic properties. One soluble factor is VDBP, which exhibits hierarchical binding with the greatest affinity for 25(OH)D_3_, the major circulating form of the vitamin; the binding affinity for D_3_ and 1*α*,25(OH)_2_D_3_ is at least ten-fold lower [19]. Studies using *VDBP* ^−/−^ mice revealed that deficiency of the binding protein resulted in lower 25(OH)D_3_ and 1*α*,25(OH)_2_D_3_ levels due to increased clearance of the unbound metabolites compared to wild-type mice [20]. After eight weeks of consuming a vitamin-D-deficient diet, *VDBP* ^−/−^ mice developed secondary hyperparathyroidism and manifested evidence of osteoporosis; neither of these signs were observed in *VDBP* ^+/+^ mice. And because VDBP purportedly facilitates delivery of activated vitamin to target tissue [21], the bound complexes could also serve as a circulating reservoir, which could contribute to the dynamic regulation of vitamin-D-related effects.

Attached to plasma membranes of vitamin-D-target tissue, a second group of proteins facilitates entry of VDBP-bound molecules [22]. However, the binding protein is not absolutely essential for internalization of the sterol. Furthermore, internal trafficking of 25(OH)D_3_ does not appear to be a random process as members of the heat shock protein 70 family act as intracellular chaperones that determine whether the metabolites are destined for enzymatic degradation or biochemical interaction with the VDR [23]. One important concept related to the latter interaction is an apparent misnomer associated with the designation “bioactive metabolite” because 1*α*,25(OH)_2_D_3_ is devoid of biological activity in the absence of the VDR [24]. In essence, the VDR is the requisite component that enables hormonal stimulus to be molecularly transcribed into hormonal effect. The significance of this principle should not be underestimated as the receptor belongs to a superfamily of nuclear factors that target genes at the transcriptional level [24]. Although a highly conserved zinc finger region essential for high-affinity binding to vitamin D response elements (VDREs) in target genes and a bridge region connecting the ligand-binding and DNA-binding domains are common to all members of the family, the VDR incorporates two unique differences [25]. First, the absence of the ligand-independent activation factor (AF)-1 motif in the N-terminus transcriptional-activating domain; second, the presence of an extraloop (not found in any other nuclear receptor) between the second and third helices in the C-terminus ligand-binding domains [26]. The functional relevance of these differences is still unclear. Even though our understanding of the receptor multiplex is incomplete, multidimensional constructs have made it possible to better appreciate and even visualize the delicate precision of the hormone-receptor interaction [27].

The primal event of hormone binding activates a sequence of events which includes induction of conformational changes in the receptor, activation of intrinsic AF-2, dissociation from heat shock proteins, phosphorylation, dimerization, and ultimately transcription [28]. Of note, the highly conserved amino acid sequence of AF-2 is especially significant as single point mutations within this short core motif have been shown to disrupt receptor transcriptional activity [29]. In addition, and unlike the homodimers formed by sex steroid hormone receptors, ligand-bound VDRs preferentially form heterodimers, particularly with one of three retinoid X receptors (RXR) [30]. 

The molecular basis by which the hormone-VDR complex interacts with nuclear transcriptional elements is extraordinarily more complicated than the foregoing events as recruitment of additional comodulators has been shown to be necessary in order to “fine tune” hormonal activity. It is important to emphasize that activation or repression of transcription depends on the balance of the recruited coregulatory components. Both types of transcriptional activity are best illustrated using 1*α*,25(OH)_2_D actions on bone although similar mechanisms may apply to the alleged non-classical effects of the vitamin. For instance, while genes that regulate calcium homeostasis (i.e., *CaT1*, calcium transport protein 1; *calbindin-D *_9K_) have been identified, the manner in which this takes place appears to depend on a number of precise interactions involving numerous coactivator complexes. Activation of targeted genes requires the presence of two chromatin remodeling complexes known as SWI/SNF-A (switching defective/sucrose non-fermenting) and SWI/SNF-B as well as coactivators of the p160 family including SRC-1, SRC-2 and SRC-3 (steroid receptor coactivator-1, 2, 3), and DRIP (vitamin D_3_ receptor-interacting protein) [31, 32]. All of the SRCs contain two activation domains, AD1 and AD2, which serve as binding sites for p300 and CARM1 (coactivator-associated arginine methyltransferase 1) [33]. The interaction between the SRC/DRIP complexes and the AF-2 domain of the VDR promotes recruitment of two epigenetic proteins to the receptor. Together with the SRC complexes, histone acetylases and methyltransferases disrupt the stability of DNA, which allow transcriptional output of the target genes.

Negative feedback is also a unique characteristic of the endocrine system. Molecular evidence suggests that part of the mechanism for self-attenuation of receptor-mediated signaling depends on the presence of corepressors. Two of the most well-known repressors of VDR-mediated transcriptional activity are NCor (nuclear corepressor) and SMRT (silencing mediator of retinoid and thyroid receptor) [34]. Notably, interactions between the VDR-NCoR and VDR-Alien complexes do not appear to involve the receptor's AF-2 domain, a finding dissimilar to the VDR-SRC coactivators mentioned previously. The implications of the different interfaces relate to the possibility that transcriptional repression may not result simply from competitive occupation of specific binding sites on DNA response elements but rather the presence of functionally different pathways for repressing transcriptional activity [35].

Another mechanism by which VDR-mediated transcriptional activity can be suppressed is somewhat of an anomaly. An endogenous protein known as VDRE-binding protein (BP) has been shown to have paradoxical effects on the regulation of DNA transcription. In the *absence* of 1*α*,25(OH)_2_D-bound VDR, VDRE-BP interacts with the same target gene promoter response elements and induces nuclear transcription [36]. However, overexpression of this binding protein can inhibit the formation of the receptor dimer-transactivating complex, thus suppressing transcriptional activity [37]. In essence, an intrinsic cellular program initiated by hormones follows a blueprint for the repetitive remodeling of chromatin, regulates the process of gene transcription, and determines specific endocrine effects on target tissue. The complexity of this conclusion can be further appreciated by the notion that vitamin D is certain to be only one functional component, albeit a very small one, of a dynamic, interactive, and adaptive process within the antimicrobial pathway.

## 5. Vitamin D and Infectious Diseases

Seldom has a compound labeled as a nutritional substance commanded as much attention as vitamin D. Aside from its role in the promotion of bone health, a measure that best supports this conclusion is the number of publications related to the extraskeletal effects of the vitamin. One area of keen interest is the pharmacologic effects and biologic relevance of the vitamin-D-dependent antimicrobial pathway.

Even before reports related to the presence of the VDR in human leukocytes were published [38], a link was loosely established between 1*α*,25(OH)_2_D and osteoclasts, which appeared at sites of active bone resorption [39]. Because cells of the monocyte/macrophage lineage are precursors of osteoclasts [40], it was hypothesized, and subsequently demonstrated that, like glucocorticoids [41], 1*α*,25(OH)_2_D could induce terminal differentiation of myeloid leukemic blasts [42, 43]. Although the underlying mechanism of their differentiating effects on both mouse and human leukemia cell lines was not known, it had been postulated that both hormones interacted with specific cytoplasmic receptors in the leukemic cells [44].

Soon thereafter, numerous publications indicated a prominent inhibitory effect of 1*α*,25(OH)_2_D on specific subsets of CD4+ T-helper cells. Not only did the hormone have the ability to suppress IL-2-mediated T-cell proliferation and function [45], the hormone could also inhibit *γ*-interferon gene transcription [46]. Notably, the suppressive effects on cell-mediated components of the immune response were somewhat disparate as inhibition of T-helper lymphocytes was much greater than the repressive effects on T-suppressor cell activity [47]. Laboratory studies subsequently demonstrated cellular components of the nonspecific immune system could also be influenced by vitamin D [48]. In two early reports, investigators showed that innate immunity was mediated, in part, by an apparent association between activation of Toll-like receptors (TLRs) and expression of antimicrobial peptides, though the biological mechanism remained elusive [49, 50].

### 5.1. Mycobacterium sp

Further investigation of monocytes stimulated with a truncated synthetic lipo-peptide derived from *Mycobacterium tuberculosis* (TLR2/1L) identified the *VDR* gene as one of two candidate genes that may be involved in the antimicrobial response of these cells. Pursuit of this finding with elegant *in vitro* studies indicated that the vitamin D pathway was the key intermediary between TLRs and innate host defense peptides [51]. In their study, Liu and colleagues demonstrated that monocytes activated by TLRs upregulated intracellular expression of both CYP27B1 and VDR. However, anticipated amplification of a number of genes downstream of the VDR, including the *cathelicidin antimicrobial peptide* (*CAMP*) gene, was not observed. Notably, the absence of *CAMP* mRNA was not due to expression of a dysfunctional VDR but rather insufficient intracellular amounts of bioactive vitamin D. Even though the latter conclusion appears inconsistent with the increased expression of 1*α*-hydroxylase (CYP27B1), its validity is supported by addition of 1*α*,25(OH)_2_D_3_, which resulted in a dose-dependent increase in expression of activated cathelicidin as well as a direct antimicrobial effect against *M. tuberculosis*.

More recently, additional effects linking VDR-signaling with innate immunity have been observed including induction of lysosomal function and *β*-defensin 4 (DEFB4) [52, 53]. Collectively, these effects take on even greater significance when coupled with the finding that the antimicrobial response was dependent on the presence of sufficient vitamin D levels. This was aptly demonstrated in a laboratory study involving monocytes cultured in medium containing either fetal calf serum (FCS) or human serum [51]. Notably, addition of TLR2/1L resulted in cathelicidin being upregulated only in monocytes maintained in media containing human serum. Because *cathelicidin* is a target gene of a functional vitamin D pathway, one plausible explanation relates to the five-fold greater amount of 25(OH)D_3_ in normal human serum compared to FCS. Validation of this belief was achieved with the demonstration that expression of cathelicidin occurred only when 25(OH)D_3_ and TLR2/1L were added simultaneously to FCS. These findings also suggested that the TLR and vitamin D pathways do not merely intersect but rather converge to fortify host defense mechanisms against microbial pathogens.

The significance of vitamin D sufficiency in laboratory models of antimicrobial response to *M. tuberculosis* has clinical correlates as well. The authors of a very recent paper sought to determine the extent to which susceptibility to active tuberculosis was associated with vitamin D deficiency among HIV-positive and HIV-negative black South Africans [54]. Exceeding the importance related to the finding that 63% of the participants were vitamin D deficient (defined as <20 ng/mL) was the apparent relationship between vitamin deficiency and increased susceptibility to active tuberculosis (TB) in both cohorts of subjects; HIV-infected (odds ratio [OR] = 5.6, 95% CI, 2.7–11.6; *P* < 0.001) and HIV-uninfected (OR = 5.2, 95% CI, 2.8–9.7; *P* < 0.001). Of interest also, reporting of new TB cases varied inversely following the three-month period with the highest and lowest mean serum 25(OH)D_3_ concentrations.

Additional *in vivo* evidence of a link between vitamin D and the innate immune response has been obtained from subjects infected with *M. leprae*, another intracellular pathogen [55]. The two forms in which leprosy present clinically enabled investigation of biological mechanisms that could account for their differences in disease severity. Molecular analysis of sample lesions from subjects with either the progressive lepromatous or the self-limited tuberculoid forms of leprosy indicated expression of a number of microribonucleic acids (miRNAs), one of which was miRNA21 (mir21). Expressed primarily in infected monocytes of those with the more severe form of the disease, mir21 was capable of downregulating TLR2/1-induced expression of *CYP27B1*. Increased expression of mir21 in monocytes cultured from lepromatous lesions was also associated with silencing of the genes responsible for encoding CAMP and DEFB4. The strong link between mir21 and the two vitamin-D-dependent antimicrobial peptides was confirmed using *mir *21^−/−^-infected monocytes with restoration of CAMP and DEFB4 expression, and TLR2/1 activity against the bacterium. These immunologic findings may partially explain the more aggressive course of the lepromatous subtype.

Even though these same investigators recently suggested that vitamin D_3_ supplementation may be immunologically advantageous to humans [55], addition of the vitamin to the treatment of leprosy has actually been advocated for approximately 100 years [56]. Nonetheless, the rationale for this recommendation may be strengthened as new information related to vitamin-D-dependent immune mechanisms is elucidated.

### 5.2. Hepatitis C Virus

A high prevalence of vitamin D deficiency among patients with severe hepatic impairment should not necessarily be surprising as the liver is the site of the initial hydroxylation reaction. This conclusion is supported by numerous investigators who reported such an association regardless of the underlying cause of liver disease [57, 58]. Even though a similar association among subjects infected with the hepatitis C virus (HCV) was not unexpected, investigators found an interesting nuance in patients with chronic HCV infection [59]. While impaired liver function is likely the primary cause of vitamin D deficiency, Lange and colleagues suggest extrahepatic factors may also be involved. Although circumstantial, the evidence is nonetheless notable. For example, a higher incidence of vitamin D deficiency was observed in patients with chronic hepatitis C, even those without fibrotic changes in the liver, compared to controls; second, published data indicate that hydroxylation of cholecalciferol can be inhibited or impaired by viral induction of inflammatory cytokines; third, the finding that circulating 25(OH)D levels increased after eradication of the virus [60].

In addition, the link between vitamin D and HCV is strengthened by correlative evidence showing a significantly higher sustained virologic response (SVR, defined as undetectable serum HCV RNA 24 weeks posttherapy) rates to anti-viral therapy among patients who did not have severe vitamin D deficiency [59, 61]. That the improved SVR may be partially due to a direct antiviral effect of vitamin D is supported by a recent publication [62]. Using a hepatoma cell line, findings related to the effect of vitamin D in HCV-infected cells are worthy of comment. First, addition of HCV to various concentrations of vitamin D-treated cells resulted in dose-dependent inhibition of HCV production. That this inhibitory effect involved the vitamin D pathway was supported by increased expression of a number of downstream targets including the *CYP27B1* gene, 1*α*,25(OH)_2_D, VDR mRNA, and 24-hydroxylase. The last finding, upregulation of *CYP24A1*, the gene encoding 24-hydroxylase, represents one potential confounding element of this study. Even though production of the bioactive metabolite is stringently controlled by hormone-induced upregulation of the gene responsible for its own catabolism and downregulation of *CYP27B1*, the only significant finding in vitamin-D-treated cells infected with HCV, was reduced expression of the catalytic enzyme. Hence, part of the inhibitory effect of vitamin D on HCV production can be explained by higher hormone concentrations (or more prolonged duration of activity) in the virus-infected cells. Another notable finding of this study was the effect of vitamin D on the innate interferon (INF) signaling pathway. In contrast to the minimal effect of both D_3_ and 1*α*,25(OH)_2_D_3_ in noninfected cells, a significant increase in *INF*-*β* gene expression was observed in virus-infected cells. Functionality of INF-*β* was correlated with induction of the INF-signaling gene *MxA*. These data provide reasonably compelling evidence that vitamin D may have a direct antiviral effect which may be partly mediated by inducing endogenous INFs.

The above data are partially counterbalanced by inconsistencies related to 25(OH)D levels and virologic response rates. Although two separate groups reported direct correlations between vitamin D levels and viral responses only in patients infected with HCV genotype 2-3 [59, 63], a third study reported the same correlation in HCV genotype 1 patients [64].

### 5.3. Human Immunodeficiency Virus (HIV)-1 

Juxtaposed to the preceding findings are data which also demonstrate that components of the adaptive immune system can be suppressed by vitamin D [52, 65–67]. As such, the disease caused by HIV-1 could also be affected. The apparent inconsistent effect on the two arms of the immune systems can be reconciled by critically examining both laboratory and epidemiological data. It is well known that resting, unstimulated T helper cells can undergo further differentiation to Th_0_, Th_1_, or Th_2_ cells. Each helper cell subset is distinguished by their characteristic pattern of lymphokines produced [68]. For example, when CD4+ cells were cocultured with 1*α*,25(OH)_2_D, the Th_1_ subset was shown to be the most sensitive to the hormone's inhibitory effect, thus suppressing IL-2 production [69]. Interestingly, by blocking antigen-presenting cell secretion of IL-12, the hormone was also able to further antagonize Th_1_ cell activation [70]. Nevertheless, these effects can be beneficial. For example, vitamin D may have therapeutic application when suppression of adaptive elements such as Th_1_ and IL-12, which have central roles in the pathogenesis of diseases such as solid organ rejection and multiple sclerosis, is desirable. In addition, the vitamin also has a critical role in *killing* intracellular pathogens by an antimicrobial process known as autophagy or programmed cell death-2 [67]. Although not restricted to *M. tuberculosis*, destruction of HIV-1 may also be induced by this process [71]. 

Even though there are no convincing data to support a *causal* relationship between vitamin D and the pathogenesis of HIV, correlative evidence of an association between vitamin deficiency and clinical progression/all-cause mortality among HIV-infected people does exist. One of the most recently published studies included 2,000 randomly selected subjects from a large international cohort of HIV-infected individuals who met specific study criteria [72]. Of the total number of subjects, 25(OH)D levels were available from 1985 persons; these subjects were segregated into three groups based on acceptable threshold values of vitamin D. For descriptive purposes and statistical analyses, the individual groups were labeled “lowest,” “middle,” and “higher,” which corresponded to 25(OH)D levels of ≤12 ng/mL, 12.1–20 ng/mL, and >20 ng/mL, respectively. Each group had similar percentages of persons based on a number of demographic characteristics. Kaplan-Meier curves of HIV infection progressing to AIDS and AIDS-related death were significantly lower among those in the middle and higher tertiles compared to subjects in the lowest vitamin D tertile. However, non-AIDS-related deaths were significantly reduced only in the higher tertile group. Another relatively large study was conducted to determine whether any correlation existed between baseline vitamin D status and three disease-related endpoints in HIV-infected pregnant women [73]. Vitamin D levels were known for 885 of the 1,078 women enrolled. In contrast to the previous study, vitamin D status was categorized as “insufficient” and “sufficient” with cut-off 25(OH)D thresholds of <32 ng/mL and ≥32 ng/mL, respectively. After a median follow-up period of nearly six years, the data indicated that pregnant women classified as having insufficient vitamin D levels had a significantly higher rate of disease progressing to stage III or higher as well as the development of severe anemia; *P* = 0.01 for both endpoints. While a relationship between vitamin D status and all-cause mortality was not observed, a significantly lower risk of dying was correlated with the highest 25(OH)D quintile approximating >55 ng/mL compared to women at the other extreme with levels <20 ng/mL.

Inherent in the above studies are a number of issues that need to be addressed. First, the relationship between low vitamin D levels and poorer clinical outcomes should be construed as correlative rather than causal. This conclusion is partially supported by methodological parameters of both studies in which 25(OH)D levels were measured in patients already infected with HIV. Hence, it is unknown to what extent the disease may have influenced vitamin D levels. Reverse-causation bias may also occur if advanced stage or progressive disease reduces sun exposure or adversely affects dietary intake, both of which could negatively impact vitamin D status. Nonetheless, and regardless of vitamin D classification, most of the subjects in both studies had early stage disease and similar CD4 T-cell counts when baseline vitamin D levels were measured. Although both of these factors weaken the notion of reverse causation, the evidence still does not establish a cause-effect relationship. Second, since the majority of subjects involved in one study were relatively young and the other restricted to mid-gestational pregnant women, both study populations may not have been truly representative of the whole cohort of HIV-infected patients. These nuances aside, it is of interest that concomitant anti-retroviral therapy (ART) did not appear to impact the results as the majority of patients in one study [72] were prescribed ART while the other not [73]. Other than drug-induced alteration of vitamin D metabolism, the effectiveness of ART subordinates a disease-related cause of vitamin D deficiency. Third, compelling data have been published that directly correlates skeletal health with circulating 25(OH)D levels [74]. In their study, Priemel and colleagues analyzed bone integrity by histomorphometric examination of 675 human bone marrow biopsy specimens. In this cohort of German adults, increased osteoid volume, surface, and thickness, which are pathologic markers of mineralization defects were not present when 25(OH)D levels exceed 30 ng/mL. The clinical relevance of this finding parallels the HIV study which used a cutoff point of ≥32 ng/mL to distinguish sufficient from insufficient vitamin D levels. However, this was not corroborated by the EuroSIDA study which showed that slower progression to AIDS or death correlated with 25(OH)D levels >12 ng/mL. Even though both assay systems used to quantify 25(OH)D levels measured only the D_3_ metabolite, it is unlikely that unmeasured 25(OH)D_2_ contributed to the notable differences which correlated vitamin D status with the observed clinical outcomes. What the findings do suggest is the vitamin D status that correlates with optimal bone health may not be the same for enhanced immune function. 

Data published within the past 15 years appear to provide provocative, though circumstantial, evidence to support the allegation that vitamin D status may influence host susceptibility to infectious diseases. The veracity of this conclusion is limited by the critical role of the VDR, a receptor that interfaces with diseases substantially different from skeletal development and bone mineralization. And because of its functional importance, herculean efforts have been made to identify *VDR* gene polymorphisms (*VDRGP*) that could have predictive or prognostic value.

## 6. *VDRGP* and Infections 

**Figure 2:**
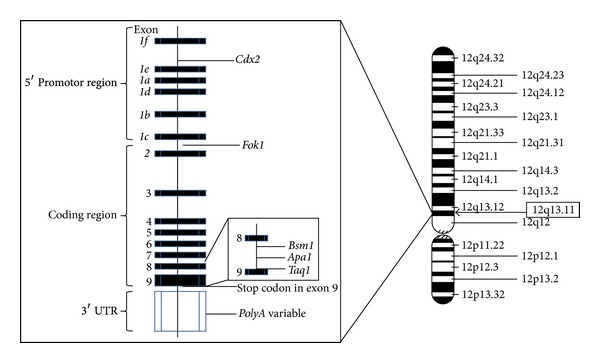
Locations of the *VDR* gene on chromosome 12 (right); and some of the currently known VDR polymorphisms (left). The gene has nine exons, eight of which (numbers 2–9) encode proteins and number one designated (*1a*–*1f*) contains six untranslated subunits.

Even though published epidemiologic data suggest that susceptibility to TB involves a complex interaction between genetic variability and environmental and cultural factors [75, 76], nutritional components, especially with regards to vitamin D, have also been reported to be associated with the risk of the disease. In a small study involving 126 *untreated* patients with TB and 116 healthy contacts who were sensitized, but did not have any clinical or radiographic evidence of the disease, vitamin D deficiency was observed in 67% and 26% of patients with active TB and healthy contacts, respectively, *P* = 0.008 [77]. These data notwithstanding, the association between vitamin D deficiency and the pathogenesis of TB is somewhat inconsistent with the observation that not all TB-infected individuals are vitamin D deficient. This finding suggests that defects in other components of the vitamin D-signaling pathway may increase the risk for, and/or progression of, the disease. One putative mechanism may involve the *VDR* gene (Figure 2). In order to address the issue whether an interaction between *VDRGP* and serum vitamin D concentrations influenced the susceptibility to the disease was also investigated. Two of the most well-studied *VDR* polymorphisms indicated that neither *Taq1* nor *Fok1* restriction enzymes alone was associated with serum vitamin D levels; however, the finding that 6.6% and 12%, (OR = 0.53; 95% CI, 0.31–0.88; *P* = 0.01), of the TB case and control patients, respectively, carried the VDR-*tt* genotype indicated that homozygosity of the *t* allele was associated with clinical resistance to the disease [78]. These findings are partially corroborated by a more recent publication involving native South Americans [79]. Among two Paraguayan populations examined, individuals harboring the VDR-TT genotype were significantly less able to mount a delayed-type hypersensitivity response to antigen challenge. 

Besides *Fok1* in the 5′ regulatory region of the *VDR* gene, single nucleotide polymorphisms (SNPs) of Cdx2 may extend to HIV and *M. tuberculosis* infections as well. The most well-characterized SNP of this transcription factor is *Cdx2*-*A* and *Cdx2*-*G* depending on whether adenine or guanine is at position −3791 base pairs relative to the transcription start site of *VDR* gene. In a study to determine the association between Cdx2 genotype and susceptibility to HIV and TB infection, Alagarasu et al. reported the frequency of the *Cdx2*-*G/A* genotype was significantly increased in HIV-positive individuals compared to healthy controls (OR = 1.89, 95% CI 1.14–3.15; *P* = 0.012) [80]. Interestingly, extended genotype frequency analysis indicated that the loose association between the *GA* haplotype and protection against HIV infection could be strengthened by the presence of two copies of that specific haplotype. 

Perhaps more notable was the observation that three restriction fragment length polymorphisms (RFLP), *Bsm*I, *Apa*I, and *Taq*I, detected in the 3′ untranslated region (UTR) of the *VDR* gene, appear to have better correlation with susceptibility to or protection against HIV infection. Genomic DNA was amplified by polymerase chain reaction for allelic discrimination of the three restriction sites. Individual RFLPs are defined by capital letters (i.e., *B, A, T*) in the absence of the restriction site; lower case letters of the same are used when the restriction sites were present. As such, the *BAt* haplotype was associated with increased susceptibility, while the *bAT* haplotype conferred protection. These findings are consistent with earlier published data showing that subjects harboring the *BAt* haplotype had decreased production of interleukin-10 (IL-10), a lymphokine with known inhibitory effects against HIV [81]. On the other hand, increased expression of 1L-10 and other antiviral chemokines coupled with downregulation of HIV coreceptor in individuals with the *bAT* haplotype are associated with defective VDR signaling and protection against HIV. Perhaps of equal importance was the association between the increased frequency of the *BAt* haplotype and the increased susceptibility to pulmonary TB in HIV-infected patients [80]. 

*VDR* gene polymorphisms continue to be relevant in order to explain the apparent disparity between vitamin action and risk of disease. One frequently occurring DNA sequence variation involves the *Bsm*1 restriction enzyme. The prevalence of the *Bsm*1 polymorphism was determined in a cohort of 185 HIV-1-seropositive individuals; the results were then correlated with disease susceptibility and progression [82]. Compared to the *VDR-Bb* and *VDR-bb* genotypes, *BB* homozygotes have identical alleles with absent restriction sites, which negatively impact the ability to synthesize the full-length VDR protein. Notably, the *VDR-BB* genotype was correlated with more rapid progression to both CD4 counts <200 cells/uL and acquired immunodeficiency syndrome (AIDS). Although the influence of the *Bsm*1 polymorphism on VDR protein function and signaling is largely unknown, the authors speculated that persons who harbored the *VDR-BB* genotype were less responsive to the immunosuppressive action of vitamin D, which could enhance Th_1_ cell activation and survival and enable a more efficient process by which the virus infected these cells.

One other interesting finding involving *Apa*1 has been reported in subjects infected with T-cell lymphotropic virus type-1 (HTLV-1) who are at risk for developing a complication known as HTLV-1-associated myelopathy (HAM). However, the observation that only a very small percentage of patients actually do suggests host factors may play an important role. In a relatively recent paper Japanese investigators genotyped the three RFLPs mentioned previously plus the *Fok*1 RFLP in patients with and without HAM [83]. Genomic analysis of 247 individuals indicated no difference in *Fok*1, *Bsm*I, and *Taq*I genotypic or allelic frequencies among the 118 subjects with HAM and 129 seropositive controls. However, a significant difference in Apa1 genotypes (i.e., *AA*,   3.9%; *Aa*,  43%; *aa*, 53.1%) was found. Upon further investigation of an additional 89 subjects with HAM (and 95 seropositive controls) a significant association was observed between females with the *AA* genotype and lower risk of developing HAM (*P* = 0.029). Although linkage disquilibrium between *Fok*1 and *Apa*1 was not apparent, cross-genotyping between the two RFLPs appeared to further clarify the protective effect, which was restricted to those who were genotypically *AA* and *Ff* for the VDR polymorphism. This finding is notable in that the frequencies of the lowest genotype was associated with the highest genotype suggesting that development of HAM would be somewhat higher were it not for the heterozygous *Fok*1 genotype. 

Leprosy susceptibility has been reported to be associated with distinct loci of chromosomes 6, 10, 17, 20 and 21. The caveat is that a specific locus may not be a risk factor for all forms of leprosy and may not confer increased risk across individuals differing in ethnicity. For example, the identification of PARK2 and PACRG at 6q25 and q26, respectively, as major leprosy risk genes in two populations, but not a third, highlights the heterogeneity of risk alleles for infectious diseases across different ethnic groups. In addition, a specific locus identified at 10p13 was confirmed as a risk factor for paucibacillary leprosy, but not overall leprosy susceptibility [84]. However, a followup study involving families from Southeast Asia and Brazil identified two SNPs in the mannose receptor 1 gene (*MRC1*) located in the 10p13 region that were associated with multibacillary leprosy and leprosy overall but not with paucibacillary disease [85]. The lack of an association with paucibacillary disease in that study suggests that the causative gene at the 10p13 locus has not yet been identified.

The *Taq*1 RFLP of the *VDR* gene has also been linked to one of two subtypes of infections caused by *M. leprae* [86]. Compared to control, homozygosity of the *t* allele was found to be significantly increased in subjects with tuberculoid leprosy (OR = 3.22, CI = 1.47–7.13; *P* = 0.001). In contrast, the lepromatous form of the disease occurred more frequently in subjects with the *TT* genotype (OR = 1.67,  CI = 1.02–2.75; *P* = 0.03) compared to controls. An extension of these disparate findings encompasses a possible, though not exclusive, association between *Taq*1 genotype and the immunomodulatory role of vitamin D. Far more complex than the implied simplicity of this conclusion is the signature adaptive immune response to the bacterial pathogen, cell-mediated in patients with tuberculoid leprosy and humoral in those with lepromatous disease. Not precisely known is how the genotypes influence the selective TH_1_ and TH_2_-type responses.

What appears to be absolutely clear (at least with regard to common *VDRGPs*) is that the amount of discordance which already exists in the only absolutely agreed upon biological role of vitamin D will likely complicate confirming the vitamin's role in other metabolic pathways as well. This conclusion is supported by studies showing interethnic differences in bone mineral density (BMD). Results of an analysis of *VDRGP* in Mexican-American Caucasian women indicated not only a change in the amino acid sequence of the VDR protein but also a correlation with BMD [87]. Using the Fok1 enzyme to cleave a 265 bp region in exon 2 resulted in three *Fok1* genotypes, *FF*, *Ff*, and *ff*. Women with the *ff* genotype had significantly lower BMD at the lumbar spine compared to women with the *FF* genotype (*P* = 0.01); and after two years of followup, BMD in the femoral neck decreased significantly in those with the *ff* genotype compared to the *FF* genotype (−4.7% versus −0.5%, *P* = 0.005). The results of this study were among the earliest to suggest that a functional *VDRGP* was apparently responsible for the observed difference. In contrast, another study involving pre- and postmenopausal Japanese women investigated whether genotyping *Fok1*, Apa1, and Taq1 alleles could also predict BMD [88]. In order to minimize potential confounding factors, the authors targeted a sample size large enough to have sufficient statistical power and a sample representative of the study population, utilized instruments assessing BMD and DNA that were valid and reliable, and controlled for factors such as anthropometric indices, race, and lifestyle. Notwithstanding these precautionary measures, their findings were inconsistent with the previous study with regards to *Fok1* polymorphism, which did not have an independent effect on BMD. 

Even more compelling evidence indicates that hypermethylated CpG (cytosine-guanine dinucleotide) regions in the *VDR* gene promotor region can, by silencing gene expression, disrupt normal vitamin D signaling. This epigenetic phenomenon may help explain the apparent resistance of tumor cells. Co-cultured with the bioactive hormone, differences in VDR transcripts and intensity of methylated CpGs were observed in breast cancer and normal breast epithelial cells [89]. An important correlative finding was that demethylation induced by inhibition of DNA methyltransferase increased expression of full-length VDR mRNA and a number of *VDRE*-genes, including 1*α*-hydroxylase, p21 (cyclin-dependent kinase inhibitor 1), and C/EBP (CCAAT-enhancer-binding proteins). The relative importance of the latter two relates to their function as tumor suppressors genes. Hence, epigenetic alterations may be another biological mechanism which contributes to vitamin D resistance in spite of adequate 25(OH)D levels. 

## 7. Conclusion

A hallmark characteristic of human infections is that development of clinical disease does not necessarily follow exposure to microbial pathogens. This feature implies that intersubject susceptibility to infectious diseases depends on multiple factors including the microorganism, environment, heredity, and host immune status. Because of the immunoregulatory role of the bioactive hormone, vitamin D may have a significant influence on the antimicrobial pathway. While published studies provide strong correlative evidence that links vitamin D status to infections, functional genomic aberrations of the vitamin D pathway are not always as clear or consistent. The reality is that DNA is a relatively simple construct; yet tethered to this scaffold is an extraordinarily dynamic genome, still malleable by the forces of mutation. This belief is especially important as gene expression patterns of microorganisms and hosts are certain to be altered continuously as an adaptive response to external stimuli. As such and as change is certain, the microbial and human genomes will continue to evolve in unpredictable ways, necessitating, and even demanding, periodic reexamination of preexisting dogma.
